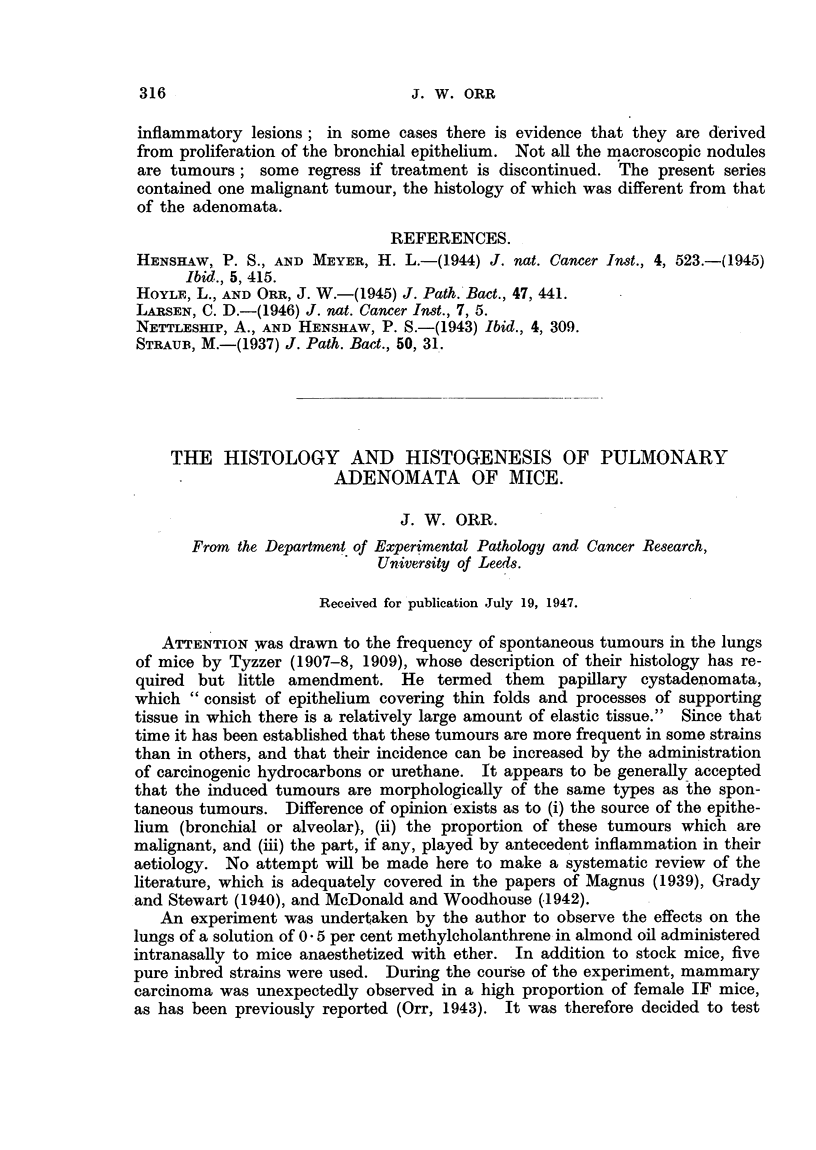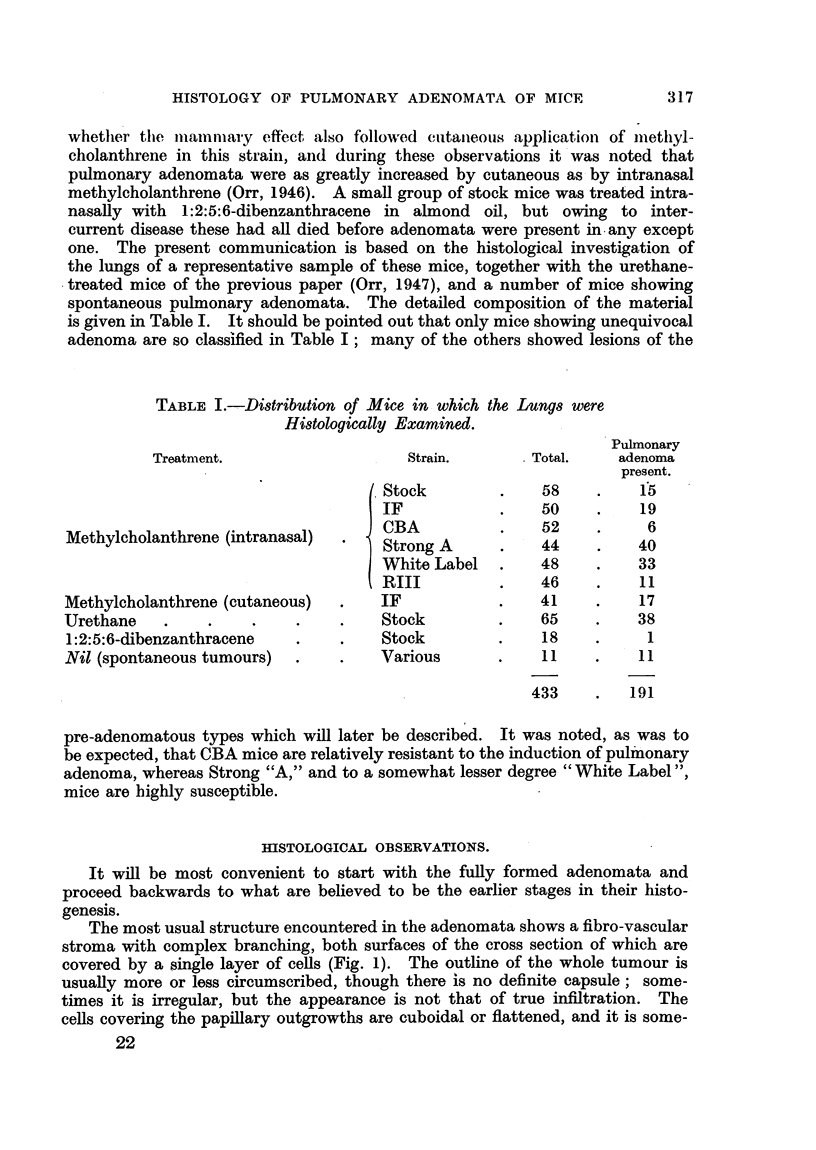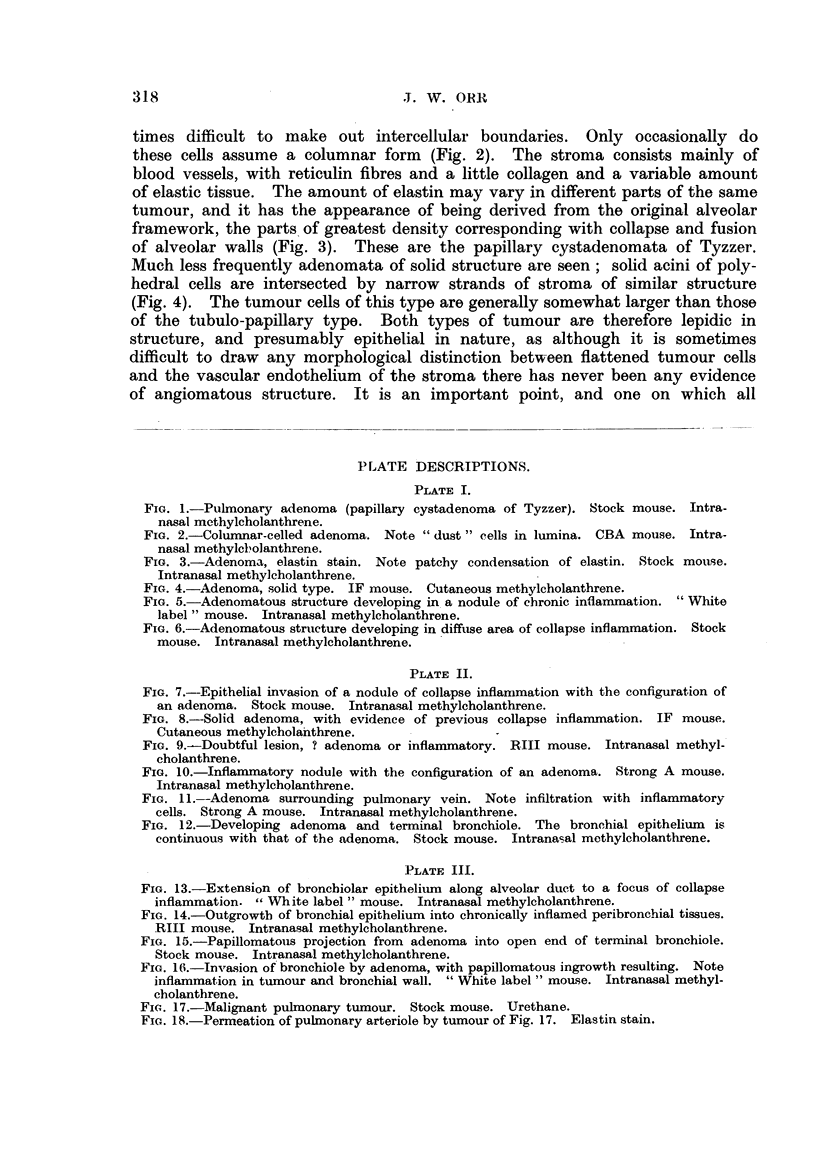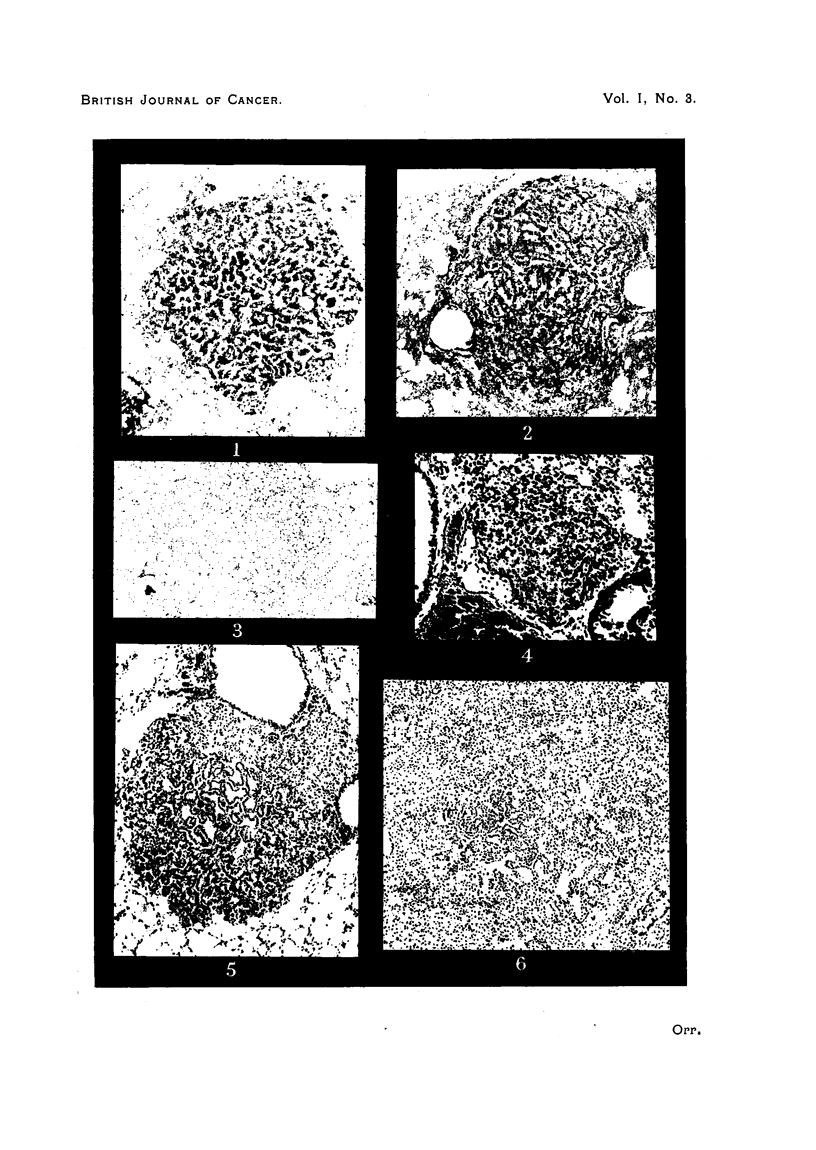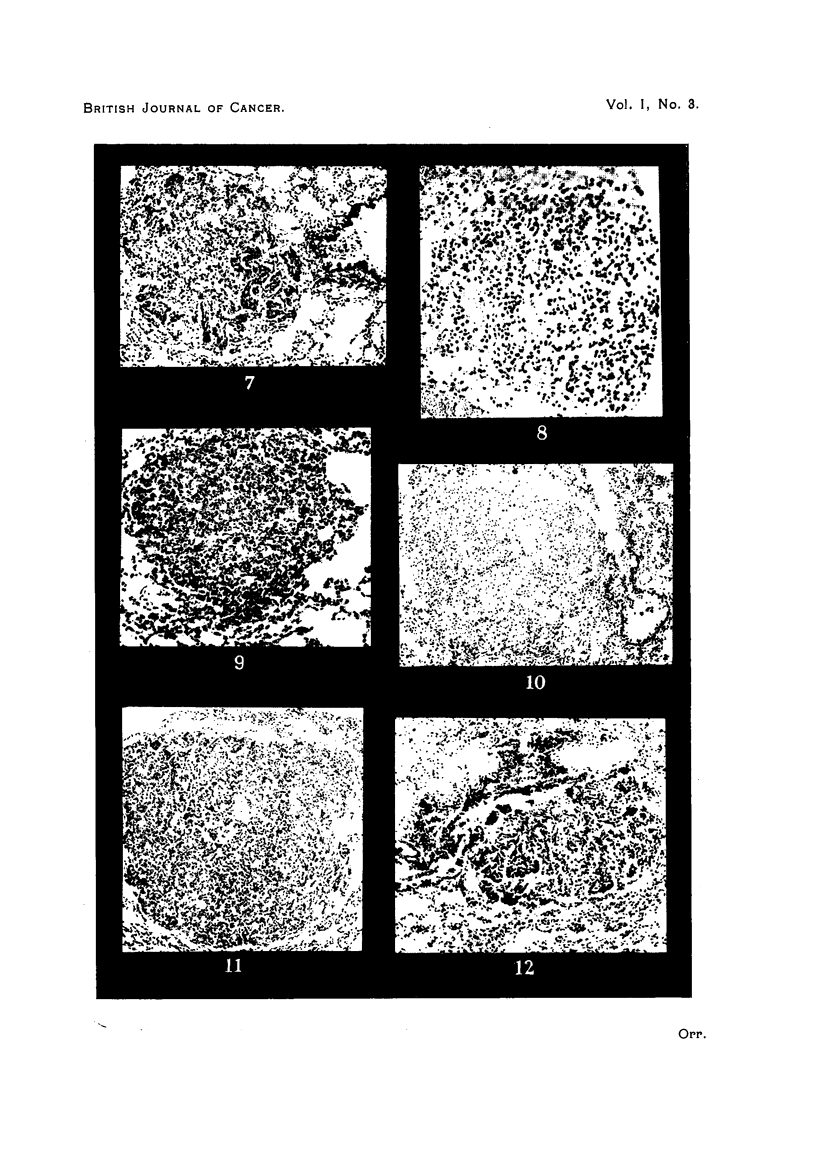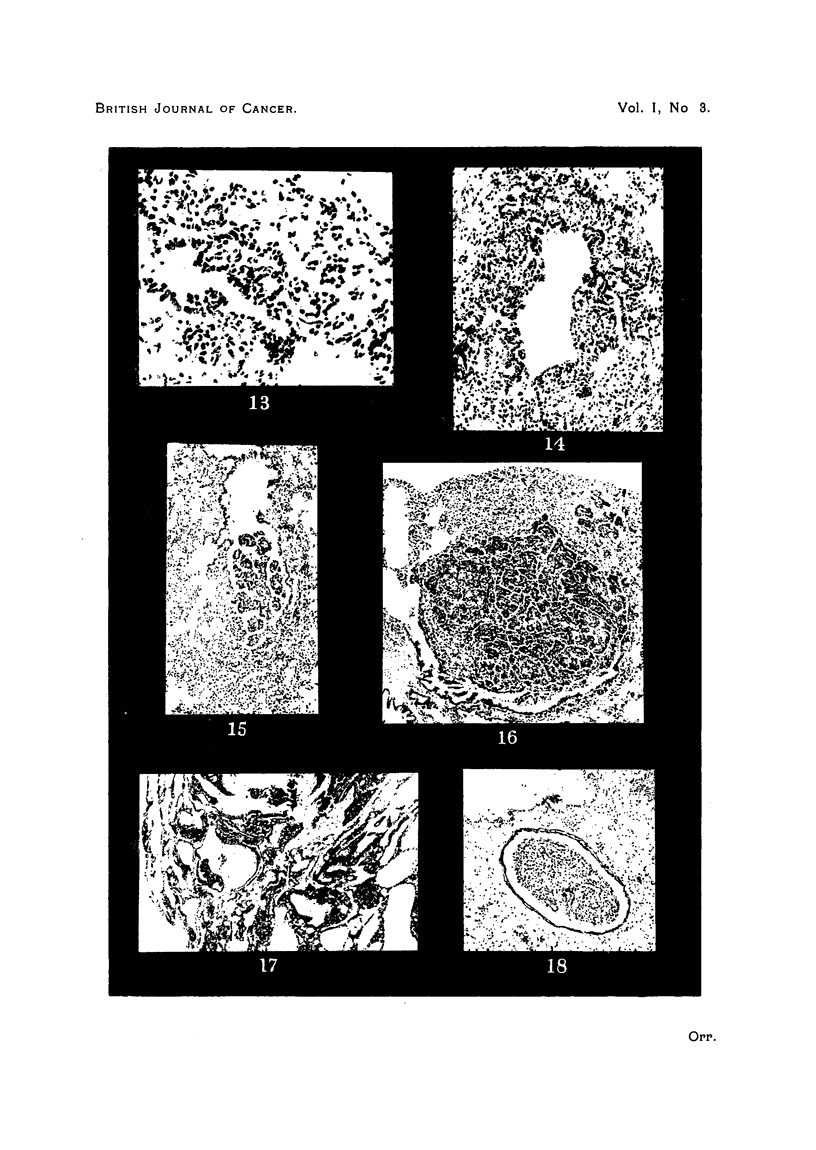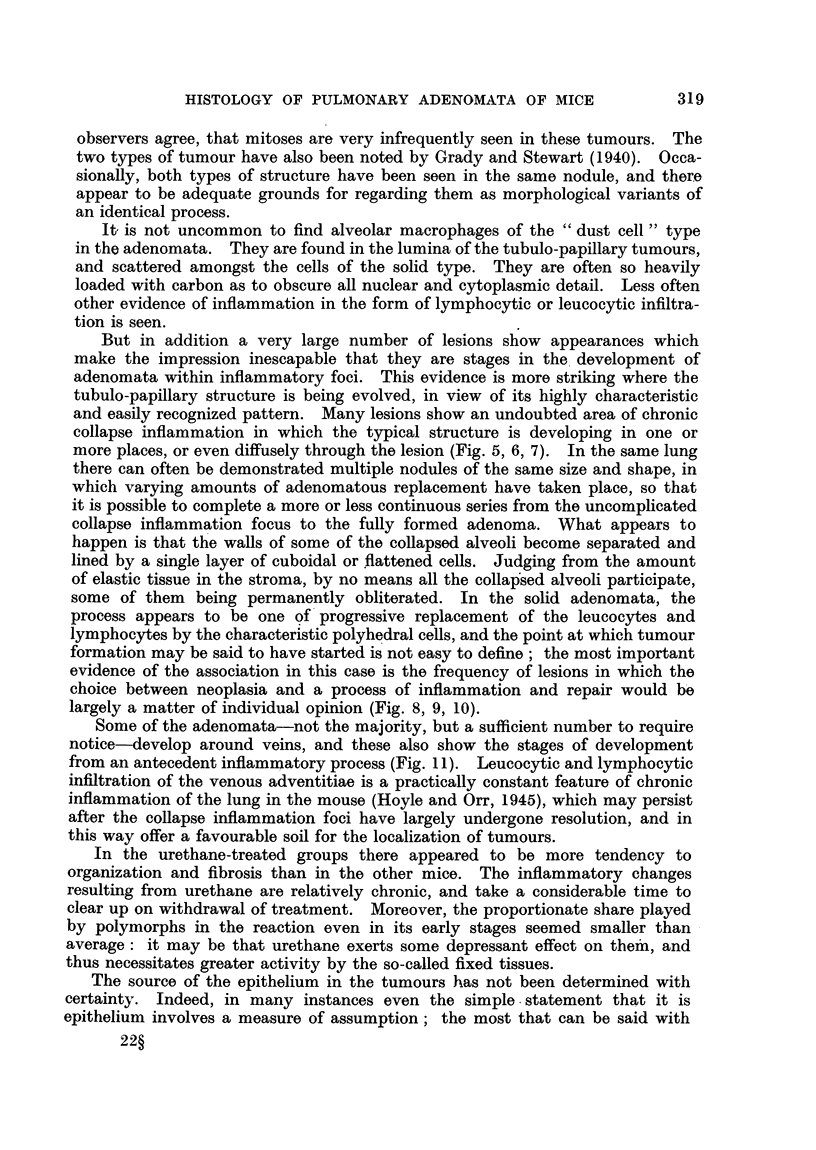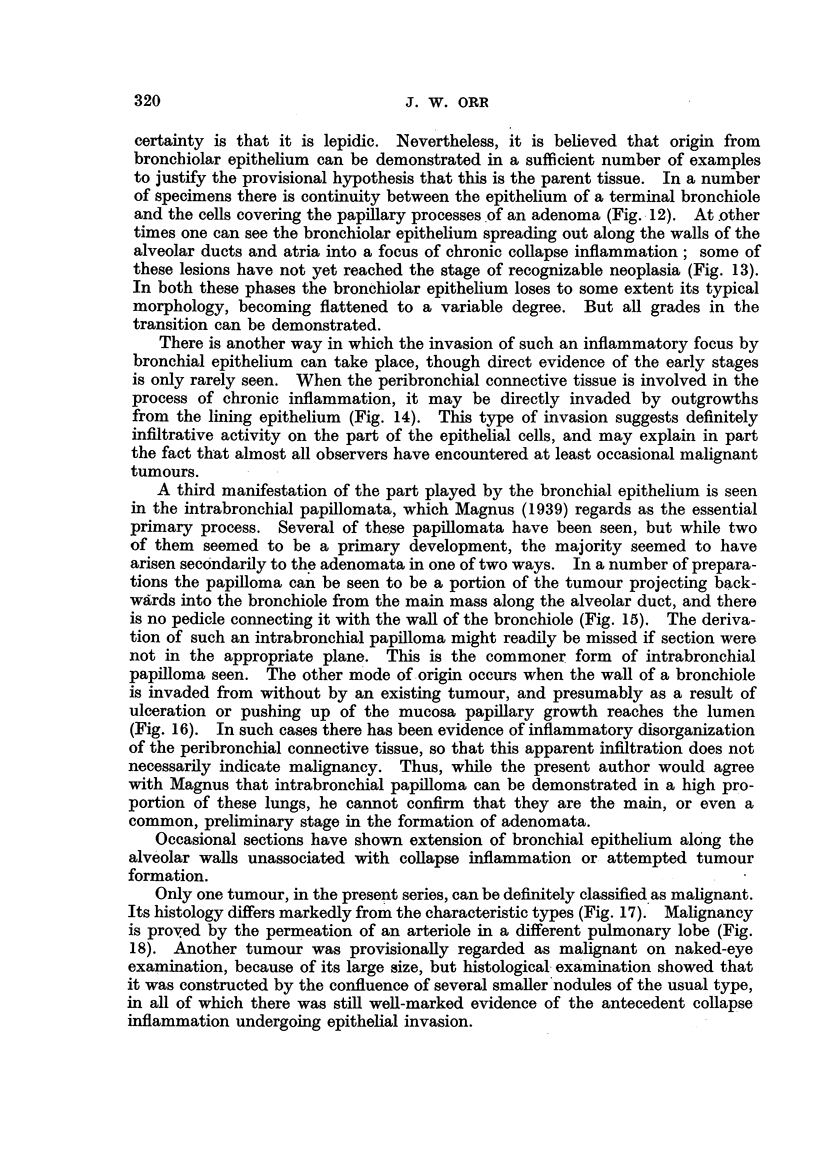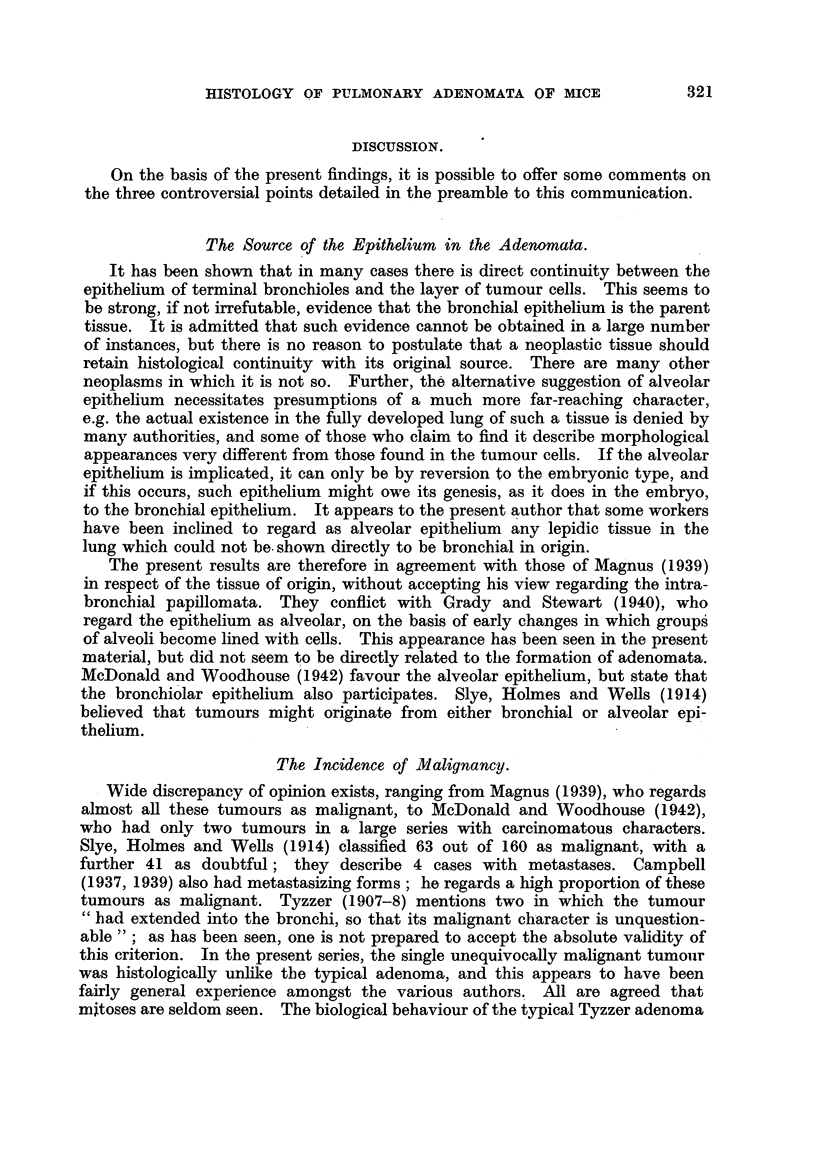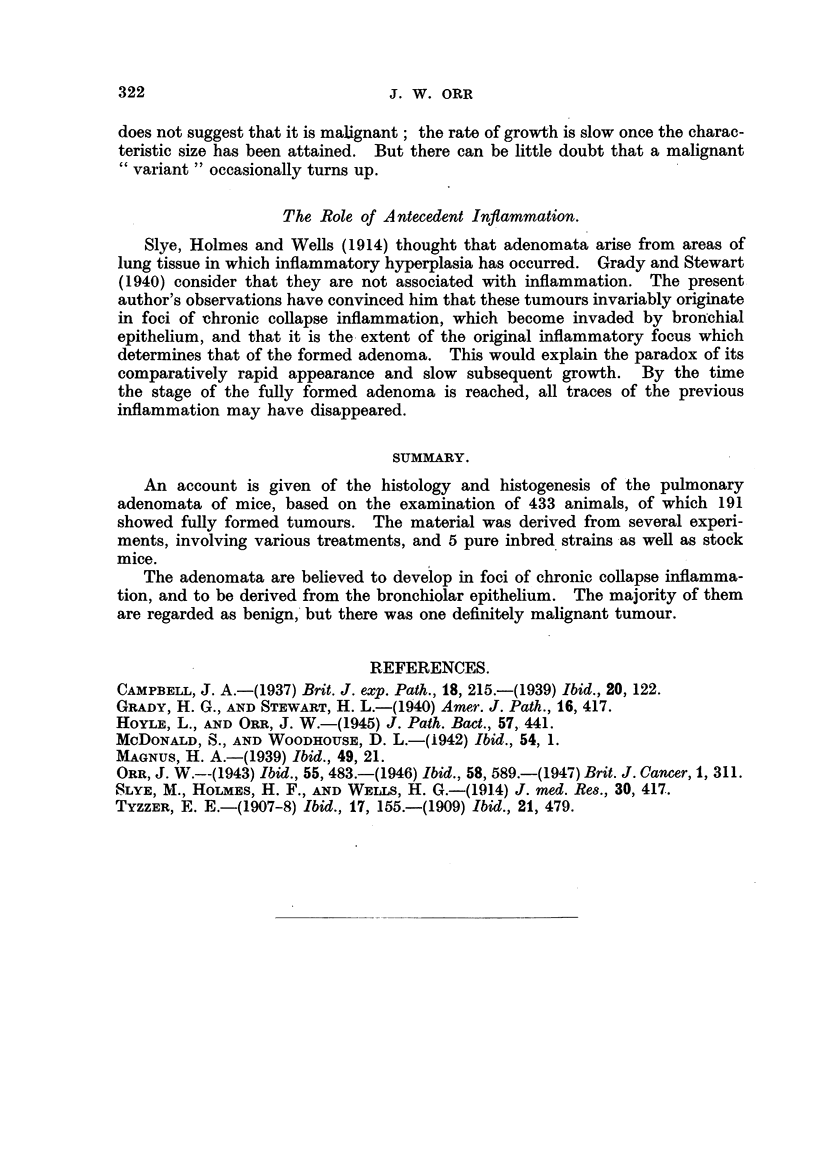# The Histology and Histogenesis of Pulmonary Adenomata of Mice

**DOI:** 10.1038/bjc.1947.27

**Published:** 1947-09

**Authors:** J. W. Orr

## Abstract

**Images:**


### THE HISTOLOGY AND HISTOGENESIS OF PULMONARY ADENOMATA OF MICE.

J. W. ORR.

*From the Department of Experimental Pathology and Cancer Research, University of Leeds.*

Received for publication July 19, 1947.

ATTENTION was drawn to the frequency of spontaneous tumours in the lungs of mice by Tyzzer (1907–8, 1909), whose description of their histology has required but little amendment. He termed them papillary cystadenomata, which "consist of epithelium covering thin folds and processes of supporting tissue in which there is a relatively large amount of elastic tissue." Since that time it has been established that these tumours are more frequent in some strains than in others, and that their incidence can be increased by the administration of carcinogenic hydrocarbons or urethane. It appears to be generally accepted that the induced tumours are morphologically of the same types as the spontaneous tumours. Difference of opinion exists as to (i) the source of the epithelium (bronchial or alveolar), (ii) the proportion of these tumours which are malignant, and (iii) the part, if any, played by antecedent inflammation in their aetiology. No attempt will be made here to make a systematic review of the literature, which is adequately covered in the papers of Magnus (1939), Grady and Stewart (1940), and McDonald and Woodhouse (1942).

An experiment was undertaken by the author to observe the effects on the lungs of a solution of 0·5 per cent methylcholanthrene in almond oil administered intranasally to mice anaesthetized with ether. In addition to stock mice, five pure inbred strains were used. During the course of the experiment, mammary carcinoma was unexpectedly observed in a high proportion of female IF mice, as has been previously reported (Orr, 1943). It was therefore decided to test whether the mammary effect also followed cutaneous application of methylcholanthrene in this strain, and during these observations it was noted that pulmonary adenomata were as greatly increased by cutaneous as by intranasal methylcholanthrene (Orr, 1946). A small group of stock mice was treated intranasally with 1:2:5:6-dibenzanthracene in almond oil, but owing to intercurrent disease these had all died before adenomata were present in any except one. The present communication is based on the histological investigation of the lungs of a representative sample of these mice, together with the urethane-treated mice of the previous paper (Orr, 1947), and a number of mice showing spontaneous pulmonary adenomata. The detailed composition of the material is given in Table I. It should be pointed out that only mice showing unequivocal adenoma are so classified in Table I; many of the others showed lesions of the

TABLE I.—*Distribution of Mice in which the Lungs were Histologically Examined.*

| Treatment. | Strain. | Total. | Pulmonary adenoma present. |
|---|---|---|---|
| Methylcholanthrene (intranasal) | Stock | 58 | 15 |
| | IF | 50 | 19 |
| | CBA | 52 | 6 |
| | Strong A | 44 | 40 |
| | White Label | 48 | 33 |
| | RIII | 46 | 11 |
| Methylcholanthrene (cutaneous) | IF | 41 | 17 |
| Urethane | Stock | 65 | 38 |
| 1:2:5:6-dibenzanthracene | Stock | 18 | 1 |
| *Nil* (spontaneous tumours) | Various | 11 | 11 |
| | | 433 | 191 |

pre-adenomatous types which will later be described. It was noted, as was to be expected, that CBA mice are relatively resistant to the induction of pulmonary adenoma, whereas Strong "A," and to a somewhat lesser degree "White Label", mice are highly susceptible.

### HISTOLOGICAL OBSERVATIONS.

It will be most convenient to start with the fully formed adenomata and proceed backwards to what are believed to be the earlier stages in their histogenesis.

The most usual structure encountered in the adenomata shows a fibro-vascular stroma with complex branching, both surfaces of the cross section of which are covered by a single layer of cells (Fig. 1). The outline of the whole tumour is usually more or less circumscribed, though there is no definite capsule; sometimes it is irregular, but the appearance is not that of true infiltration. The cells covering the papillary outgrowths are cuboidal or flattened, and it is sometimes difficult to make out intercellular boundaries. Only occasionally do these cells assume a columnar form (Fig. 2). The stroma consists mainly of blood vessels, with reticulin fibres and a little collagen and a variable amount of elastic tissue. The amount of elastin may vary in different parts of the same tumour, and it has the appearance of being derived from the original alveolar framework, the parts of greatest density corresponding with collapse and fusion of alveolar walls (Fig. 3). These are the papillary cystadenomata of Tyzzer. Much less frequently adenomata of solid structure are seen ; solid acini of polyhedral cells are intersected by narrow strands of stroma of similar structure (Fig. 4). The tumour cells of this type are generally somewhat larger than those of the tubulo-papillary type. Both types of tumour are therefore lepidic in structure, and presumably epithelial in nature, as although it is sometimes difficult to draw any morphological distinction between flattened tumour cells and the vascular endothelium of the stroma there has never been any evidence of angiomatous structure. It is an important point, and one on which all

---

### PLATE DESCRIPTIONS.

### PLATE I.

FIG. 1.—Pulmonary adenoma (papillary cystadenoma of Tyzzer). Stock mouse. Intranasal methylcholanthrene.
FIG. 2.—Columnar-celled adenoma. Note " dust " cells in lumina. CBA mouse. Intranasal methylcholanthrene.
FIG. 3.—Adenoma, elastin stain. Note patchy condensation of elastin. Stock mouse. Intranasal methylcholanthrene.
FIG. 4.—Adenoma, solid type. IF mouse. Cutaneous methylcholanthrene.
FIG. 5.—Adenomatous structure developing in a nodule of chronic inflammation. " White label " mouse. Intranasal methylcholanthrene.
FIG. 6.—Adenomatous structure developing in diffuse area of collapse inflammation. Stock mouse. Intranasal methylcholanthrene.

### PLATE II.

FIG. 7.—Epithelial invasion of a nodule of collapse inflammation with the configuration of an adenoma. Stock mouse. Intranasal methylcholanthrene.
FIG. 8.—Solid adenoma, with evidence of previous collapse inflammation. IF mouse. Cutaneous methylcholanthrene.
FIG. 9.—Doubtful lesion, ? adenoma or inflammatory. RIII mouse. Intranasal methylcholanthrene.
FIG. 10.—Inflammatory nodule with the configuration of an adenoma. Strong A mouse. Intranasal methylcholanthrene.
FIG. 11.—Adenoma surrounding pulmonary vein. Note infiltration with inflammatory cells. Strong A mouse. Intranasal methylcholanthrene.
FIG. 12.—Developing adenoma and terminal bronchiole. The bronchial epithelium is continuous with that of the adenoma. Stock mouse. Intranasal methylcholanthrene.

### PLATE III.

FIG. 13.—Extension of bronchiolar epithelium along alveolar duct to a focus of collapse inflammation. " White label " mouse. Intranasal methylcholanthrene.
FIG. 14.—Outgrowth of bronchial epithelium into chronically inflamed peribronchial tissues. RIII mouse. Intranasal methylcholanthrene.
FIG. 15.—Papillomatous projection from adenoma into open end of terminal bronchiole. Stock mouse. Intranasal methylcholanthrene.
FIG. 16.—Invasion of bronchiole by adenoma, with papillomatous ingrowth resulting. Note inflammation in tumour and bronchial wall. " White label " mouse. Intranasal methylcholanthrene.
FIG. 17.—Malignant pulmonary tumour. Stock mouse. Urethane.
FIG. 18.—Permeation of pulmonary arteriole by tumour of Fig. 17. Elastin stain.

1
2
3
4
5
6

7

8

9

10

11

12

13

14

15

16

17

18

Orr.

observers agree, that mitoses are very infrequently seen in these tumours. The two types of tumour have also been noted by Grady and Stewart (1940). Occasionally, both types of structure have been seen in the same nodule, and there appear to be adequate grounds for regarding them as morphological variants of an identical process.

It is not uncommon to find alveolar macrophages of the " dust cell " type in the adenomata. They are found in the lumina of the tubulo-papillary tumours, and scattered amongst the cells of the solid type. They are often so heavily loaded with carbon as to obscure all nuclear and cytoplasmic detail. Less often other evidence of inflammation in the form of lymphocytic or leucocytic infiltration is seen.

But in addition a very large number of lesions show appearances which make the impression inescapable that they are stages in the development of adenomata within inflammatory foci. This evidence is more striking where the tubulo-papillary structure is being evolved, in view of its highly characteristic and easily recognized pattern. Many lesions show an undoubted area of chronic collapse inflammation in which the typical structure is developing in one or more places, or even diffusely through the lesion (Fig. 5, 6, 7). In the same lung there can often be demonstrated multiple nodules of the same size and shape, in which varying amounts of adenomatous replacement have taken place, so that it is possible to complete a more or less continuous series from the uncomplicated collapse inflammation focus to the fully formed adenoma. What appears to happen is that the walls of some of the collapsed alveoli become separated and lined by a single layer of cuboidal or flattened cells. Judging from the amount of elastic tissue in the stroma, by no means all the collapsed alveoli participate, some of them being permanently obliterated. In the solid adenomata, the process appears to be one of progressive replacement of the leucocytes and lymphocytes by the characteristic polyhedral cells, and the point at which tumour formation may be said to have started is not easy to define ; the most important evidence of the association in this case is the frequency of lesions in which the choice between neoplasia and a process of inflammation and repair would be largely a matter of individual opinion (Fig. 8, 9, 10).

Some of the adenomata—not the majority, but a sufficient number to require notice—develop around veins, and these also show the stages of development from an antecedent inflammatory process (Fig. 11). Leucocytic and lymphocytic infiltration of the venous adventitiae is a practically constant feature of chronic inflammation of the lung in the mouse (Hoyle and Orr, 1945), which may persist after the collapse inflammation foci have largely undergone resolution, and in this way offer a favourable soil for the localization of tumours.

In the urethane-treated groups there appeared to be more tendency to organization and fibrosis than in the other mice. The inflammatory changes resulting from urethane are relatively chronic, and take a considerable time to clear up on withdrawal of treatment. Moreover, the proportionate share played by polymorphs in the reaction even in its early stages seemed smaller than average : it may be that urethane exerts some depressant effect on them, and thus necessitates greater activity by the so-called fixed tissues.

The source of the epithelium in the tumours has not been determined with certainty. Indeed, in many instances even the simple statement that it is epithelium involves a measure of assumption ; the most that can be said with certainty is that it is lepidic. Nevertheless, it is believed that origin from bronchiolar epithelium can be demonstrated in a sufficient number of examples to justify the provisional hypothesis that this is the parent tissue. In a number of specimens there is continuity between the epithelium of a terminal bronchiole and the cells covering the papillary processes of an adenoma (Fig. 12). At other times one can see the bronchiolar epithelium spreading out along the walls of the alveolar ducts and atria into a focus of chronic collapse inflammation ; some of these lesions have not yet reached the stage of recognizable neoplasia (Fig. 13). In both these phases the bronchiolar epithelium loses to some extent its typical morphology, becoming flattened to a variable degree. But all grades in the transition can be demonstrated.

There is another way in which the invasion of such an inflammatory focus by bronchial epithelium can take place, though direct evidence of the early stages is only rarely seen. When the peribronchial connective tissue is involved in the process of chronic inflammation, it may be directly invaded by outgrowths from the lining epithelium (Fig. 14). This type of invasion suggests definitely infiltrative activity on the part of the epithelial cells, and may explain in part the fact that almost all observers have encountered at least occasional malignant tumours.

A third manifestation of the part played by the bronchial epithelium is seen in the intrabronchial papillomata, which Magnus (1939) regards as the essential primary process. Several of these papillomata have been seen, but while two of them seemed to be a primary development, the majority seemed to have arisen secondarily to the adenomata in one of two ways. In a number of preparations the papilloma can be seen to be a portion of the tumour projecting backwards into the bronchiole from the main mass along the alveolar duct, and there is no pedicle connecting it with the wall of the bronchiole (Fig. 15). The derivation of such an intrabronchial papilloma might readily be missed if section were not in the appropriate plane. This is the commoner form of intrabronchial papilloma seen. The other mode of origin occurs when the wall of a bronchiole is invaded from without by an existing tumour, and presumably as a result of ulceration or pushing up of the mucosa papillary growth reaches the lumen (Fig. 16). In such cases there has been evidence of inflammatory disorganization of the peribronchial connective tissue, so that this apparent infiltration does not necessarily indicate malignancy. Thus, while the present author would agree with Magnus that intrabronchial papilloma can be demonstrated in a high proportion of these lungs, he cannot confirm that they are the main, or even a common, preliminary stage in the formation of adenomata.

Occasional sections have shown extension of bronchial epithelium along the alveolar walls unassociated with collapse inflammation or attempted tumour formation.

Only one tumour, in the present series, can be definitely classified as malignant. Its histology differs markedly from the characteristic types (Fig. 17). Malignancy is proved by the permeation of an arteriole in a different pulmonary lobe (Fig. 18). Another tumour was provisionally regarded as malignant on naked-eye examination, because of its large size, but histological examination showed that it was constructed by the confluence of several smaller nodules of the usual type, in all of which there was still well-marked evidence of the antecedent collapse inflammation undergoing epithelial invasion.

### DISCUSSION.

On the basis of the present findings, it is possible to offer some comments on the three controversial points detailed in the preamble to this communication.

### *The Source of the Epithelium in the Adenomata.*

It has been shown that in many cases there is direct continuity between the epithelium of terminal bronchioles and the layer of tumour cells. This seems to be strong, if not irrefutable, evidence that the bronchial epithelium is the parent tissue. It is admitted that such evidence cannot be obtained in a large number of instances, but there is no reason to postulate that a neoplastic tissue should retain histological continuity with its original source. There are many other neoplasms in which it is not so. Further, the alternative suggestion of alveolar epithelium necessitates presumptions of a much more far-reaching character, e.g. the actual existence in the fully developed lung of such a tissue is denied by many authorities, and some of those who claim to find it describe morphological appearances very different from those found in the tumour cells. If the alveolar epithelium is implicated, it can only be by reversion to the embryonic type, and if this occurs, such epithelium might owe its genesis, as it does in the embryo, to the bronchial epithelium. It appears to the present author that some workers have been inclined to regard as alveolar epithelium any lepidic tissue in the lung which could not be shown directly to be bronchial in origin.

The present results are therefore in agreement with those of Magnus (1939) in respect of the tissue of origin, without accepting his view regarding the intra-bronchial papillomata. They conflict with Grady and Stewart (1940), who regard the epithelium as alveolar, on the basis of early changes in which groups of alveoli become lined with cells. This appearance has been seen in the present material, but did not seem to be directly related to the formation of adenomata. McDonald and Woodhouse (1942) favour the alveolar epithelium, but state that the bronchiolar epithelium also participates. Slye, Holmes and Wells (1914) believed that tumours might originate from either bronchial or alveolar epithelium.

### *The Incidence of Malignancy.*

Wide discrepancy of opinion exists, ranging from Magnus (1939), who regards almost all these tumours as malignant, to McDonald and Woodhouse (1942), who had only two tumours in a large series with carcinomatous characters. Slye, Holmes and Wells (1914) classified 63 out of 160 as malignant, with a further 41 as doubtful; they describe 4 cases with metastases. Campbell (1937, 1939) also had metastasizing forms; he regards a high proportion of these tumours as malignant. Tyzzer (1907–8) mentions two in which the tumour "had extended into the bronchi, so that its malignant character is unquestionable"; as has been seen, one is not prepared to accept the absolute validity of this criterion. In the present series, the single unequivocally malignant tumour was histologically unlike the typical adenoma, and this appears to have been fairly general experience amongst the various authors. All are agreed that mitoses are seldom seen. The biological behaviour of the typical Tyzzer adenoma does not suggest that it is malignant ; the rate of growth is slow once the characteristic size has been attained. But there can be little doubt that a malignant " variant " occasionally turns up.

### *The Role of Antecedent Inflammation.*

Slye, Holmes and Wells (1914) thought that adenomata arise from areas of lung tissue in which inflammatory hyperplasia has occurred. Grady and Stewart (1940) consider that they are not associated with inflammation. The present author's observations have convinced him that these tumours invariably originate in foci of chronic collapse inflammation, which become invaded by bronchial epithelium, and that it is the extent of the original inflammatory focus which determines that of the formed adenoma. This would explain the paradox of its comparatively rapid appearance and slow subsequent growth. By the time the stage of the fully formed adenoma is reached, all traces of the previous inflammation may have disappeared.

### SUMMARY.

An account is given of the histology and histogenesis of the pulmonary adenomata of mice, based on the examination of 433 animals, of which 191 showed fully formed tumours. The material was derived from several experiments, involving various treatments, and 5 pure inbred strains as well as stock mice.

The adenomata are believed to develop in foci of chronic collapse inflammation, and to be derived from the bronchiolar epithelium. The majority of them are regarded as benign, but there was one definitely malignant tumour.

### REFERENCES.

Campbell, J. A.—(1937) *Brit. J. exp. Path.*, **18**, 215.—(1939) *Ibid.*, **20**, 122.
Grady, H. G., and Stewart, H. L.—(1940) *Amer. J. Path.*, **16**, 417.
Hoyle, L., and Orr, J. W.—(1945) *J. Path. Bact.*, **57**, 441.
McDonald, S., and Woodhouse, D. L.—(1942) *Ibid.*, **54**, 1.
Magnus, H. A.—(1939) *Ibid.*, **49**, 21.
Orr, J. W.—(1943) *Ibid.*, **55**, 483.—(1946) *Ibid.*, **58**, 589.—(1947) *Brit. J. Cancer*, **1**, 311.
Slye, M., Holmes, H. F., and Wells, H. G.—(1914) *J. med. Res.*, **30**, 417.
Tyzzer, E. E.—(1907-8) *Ibid.*, **17**, 155.—(1909) *Ibid.*, **21**, 479.